# Sleep disruptions and associated risk factors among Iranians during Ramadan amid COVID-19 pandemic: A nationwide cross-sectional study

**DOI:** 10.1097/MD.0000000000038454

**Published:** 2024-05-31

**Authors:** Sohrab Amiri, Mohammad Pourfridoni, Reza Heidari-Soureshjani, Mitra Sotoudeh, MoezAlIslam E. Faris, Amna G. Albalushi, Fatima Alsaedi, Sajjad Ahmed Khan, Moien A.B. Khan

**Affiliations:** a Medicine, Quran and Hadith Research Center, Baqiyatallah University of Medical Sciences, Tehran, Iran; b Student Research Committee, Jiroft University of Medical Sciences, Jiroft, Iran; c School of Nursing Midwifery, Tehran University of Medical Sciences, Tehran, Iran; d Iranshahr University of Medical Sciences, Iranshahr, Iran; e Department of Clinical Nutrition and Dietetics, College of Health Sciences, Sharjah Institute for Medical and Health Sciences (RIMHS), University of Sharjah, Sharjah, United Arab Emirates; f Ambulatory Health Services, Abu Dhabi, United Arab Emirates; g Birat Medical College Teaching Hospital, Morang, Nepal; h Department of Family Medicine, Health and wellness research group, College of Medicine and Health Sciences, United Arab Emirates University, Al-Ain, United Arab Emirates.

**Keywords:** lifestyle choices, Ramadan fasting, sleep disruptions, sociodemographic factors

## Abstract

The global COVID-19 pandemic has disrupted daily routines and heightened stress levels worldwide, impacting various aspects of health, including sleep. This cross-sectional study conducted in Iran aimed to investigate the impact of Ramadan Intermittent Fasting (RIF) during the pandemic on sleep disturbances and associated risk factors in adults aged ≥ 18 years. Data was collected from Iranian participants observing RIF. A structured electronic questionnaire, translated into Persian, gathered information on sociodemographics, dietary habits, sleep parameters, physical activity, and more. The study assessed sleep quality, duration, and disturbances and conducted a thorough analysis to identify risk factors associated with sleep disruptions. The study revealed that 48% of the participants experienced sleep disturbances during RIF, with 21% reporting poor sleep quality and 46% having unusual sleep duration. Various factors were associated with an elevated risk of sleep disruptions, including body mass index, place of residence, income levels, cohabitation status, family history of obesity, hypertension, diabetes, heart disease, personal history of obesity, weight fluctuations, Shisha smoking, and unemployment. The results highlight the critical importance of health promotion strategies tailored to cultural contexts. This study advocates for enhanced health education initiatives focusing on sleep hygiene and stress management, especially during periods of significant lifestyle alterations like Ramadan amid a global pandemic. Such initiatives are vital in mitigating sleep disturbances and promoting overall well-being in populations facing unique health challenges.

## 1. Introduction

Sleep is an essential component of health and well-being, having implications across the physical, cognitive, emotional, and immunological functions.^[1,2]^ The corona virus disease (COVID-19) pandemic, with its unique stressors like virus fear, financial uncertainty, and social isolation, has notably impacted sleep patterns worldwide.^[3,4]^ Previous studies have underscored the prevalence of sleep disorders during this period, highlighting the influence of global and cultural factors in understanding these disturbances.^[5,6]^ Another study emphasizes the importance of sleep in preserving life and improving our overall quality of life.^[7]^ However, the delicate balance of sleep can be upset by a variety of things, including pressures and lifestyle changes. The tremendous challenges posed by the COVID-19 epidemic have transformed practically every aspect of our lives, affecting individuals all around the world.

Stress, a common reaction to difficult circumstances, has been shown to have a major impact on sleep patterns and quality. Stressors caused by the pandemic, combined with measures like lockdowns and remote work, have altered our sleep cycles.^[8]^ Understanding the incidence and underlying reasons for these sleep disturbances during this period is critical to understanding the pandemic’s influence on human health and well-being.

The Stress-Adaptation Model is one psychological hypothesis that may shed light on these sleep disturbances.^[9]^ According to this theory stressors like the COVID-19 pandemic might cause psychological and physical reactions that affect sleep habits.^[10]^ The pandemic’s uncertainties, concerns, and disturbances in social and economic dynamics may have increased stress levels.^[11]^ According to the Stress-Adaptation Model, greater stress might appear as sleep issues when our bodies adapt to new circumstances.

Furthermore, our research is unique in that it takes place during Ramadan, a distinct month that requires healthy adult Muslims to fast from dawn to sunset. Dietary habits, meal timings, and sleep cycles frequently shift dramatically during this time.^[5,12]^ The co-occurrence of the COVID-19 epidemic and Ramadan provides an excellent chance to thoroughly explore sleep disturbances and their related risk factors.^[13]^ Sleep disturbances, poor sleep quality, and uneven sleep duration are examples of these disruptions. Understanding how a global health crisis intersects with a religious fasting month to influence these sleep changes sheds light on the intricate interplay of health, culture, and environmental factors.

Like many others, Iranians were forced to adjust their health and lifestyle as a result of the pandemic.^[14]^ In this descriptive cross-sectional study, we surveyed participants to examine their sleep habits before and during Ramadan, within the context of the COVID-19 pandemic. The aim was to conduct a comparative analysis to uncover the potential effects of the pandemic and associated lifestyle adjustments on sleep patterns, thereby shedding light on how these significant behavioral shifts have influenced sleep behaviors. The timing of the COVID-19 epidemic and Ramadan in 2020 is especially relevant in Iran since Ramadan bears tremendous cultural significance.^[15]^ Our study, conducted during the pandemic and Ramadan, aims to contribute to the understanding of health implications related to these concurrent events, focusing on sleep patterns.

In summary, the importance of sleep in health assessment cannot be over-emphasized. Sleep disturbances can lead to a variety of health problems, including chronic diseases, cognitive deficits, emotional disorders, and decreased immunological function. Our research aims to provide a more nuanced knowledge of sleep patterns during Ramadan, which coincides with the COVID-19 epidemic, therefore adding to a more comprehensive understanding of sleep interruptions during times of exceptional global and cultural significance.

Existing research also emphasizes the impact of demographic characteristics, lifestyle choices, and health problems on sleep.^[16,17]^ With this context in mind, our thorough study investigates sleep disturbances and the risk variables linked with them during COVID-19 Ramadan in Iran. In this research, the first aim was to investigate sleep disturbances, sleep quality, and sleep duration prevalence in the Iranian population during COVID-19. We also investigated gender differences in sleep disruption prevalence and examined how marital status, work status, and living region influence sleep patterns. In addition, we looked into the socioeconomic, lifestyle, and family/personal health history aspects that contribute to sleep interruptions, poor sleep quality, and irregular sleep length. Protective variables like self-reported health and a healthy diet are also being investigated. Furthermore, our research also looks into the effects of age on sleep duration and the relationship between BMI and sleep disorders. These multifaceted aims collectively form the foundation of our comprehensive exploration into sleepdisturbances and their associated risk factors during the COVID-19 pandemic and Ramadan observance in Iran.

## 2. Methods

### 2.1. Study design

This research employed a cross-sectional, observational design to investigate the impact of Ramadan intermittent fasting (RIF) on lifestyle and dietary changes in adult Muslims residing in Iran during the COVID-19 pandemic. Data collection commenced on May 10, 2021, corresponding to the 27th Ramadan month in 1442 Hijri, and concluded on June 10, 2021, marking the 29th Shawwal 1442 Hijri. The study focused on adult Muslims aged ≥ 18 years who observed RIF while residing in Iran. Individuals with a history of diagnosed or treated mental health problems, those following specific diets, and individuals engaged in shift work were excluded from participation at the onset of the electronic questionnaire. Exclusion criteria were applied based on responses to a specific question in the Google Form, which asked participants whether they had been diagnosed with mental health issues or were undergoing treatment for mental health conditions. Those affirming either condition were excluded to control for potential confounding variables affecting sleep disturbances.

### 2.2. Data collection

This study was initiated and overseen by a team of researchers based in the United Arab Emirates (UAE). To gather data, a snowball sampling approach was employed, with research collaborators recruited from various regions in Iran. Collaborators were tasked with utilizing web-based platforms such as emails, WhatsApp, and Facebook to disseminate survey forms and unique links for data collection. Data from multiple sources were consolidated upon completion of data collection, effectively ensuring widespread distribution of the survey questionnaire, even during periods of pandemic-related lockdowns.

### 2.3. Sample size calculation

To determine the required sample size, an a priori G* power estimate was conducted for Iran, utilizing one-tailed Student’s *t* tests and a bivariate correlation analysis model.^[18]^ The analysis aimed to identify the necessary sample size, given an estimated effect size of 0.2, an alpha error of 0.05, and a desired power of 0.90. The findings indicated a minimum requirement of 207 participants from Iran to achieve the study’s objectives.

### 2.4. Ethical considerations

This study adhered to ethical principles outlined in the Declaration of Helsinki.^[19]^ Ethical approvals were obtained from the Social Sciences Research Ethics Committee of the United Arab Emirates University (Approval number ERS 2021-7308) and Tehran University of Medical Sciences (Approval number IR.TUMS.FNM.REC.1400.022). Before participation, all participants were fully informed about the study’s objectives and procedures, and informed consent was obtained. No financial or non-financial incentives were provided to participants for their involvement.

### 2.5. Questionnaire development and translation

A structured, self-administered electronic questionnaire was developed to collect data on demographic information, dietary intake, eating habits, sleep parameters, physical activity, computer use for work and study, and smoking habits during RIF.^[20–24]^ The questionnaire was distributed online using web-based platforms, and it was translated into the Persian language. The translation process followed established guidelines for translation and cultural adaptation.^[25]^ Forward and backward translations were conducted, and the questionnaire underwent pilot testing with 30 participants to ensure clarity and comprehensibility.

### 2.6. Data cleaning

To maintain data quality, response options with similar meanings were standardized. Participants who omitted crucial questions were excluded from the analysis. Non-fasting individuals, including those who reported zero fasting days, were also excluded. Body Mass Index (BMI) was calculated using self-reported height and weight and categorized based on WHO definitions.^[26]^ Dietary pattern questions were reclassified, and participants below the age of 18 and those with abnormal values were excluded from the analysis.

### 2.7. Questionnaire measures

The questionnaire gathered sociodemographic information, including age, gender, residence, nationality, marital status, living area, household income, living conditions, education level, and the number of fasting days. Total household income was categorized into quintiles, and participants assessed their economic status relative to their community. Smoking behaviors (cigarette and shisha) were assessed before and during RIF. Physical activity levels were evaluated, and self-reported energy levels, computer usage, and screen time (TV, Computer, Entertainment) were recorded. Participants’ self-reported height and weight were used to calculate BMI. Dietary information was collected, including self-reported dietary quality during RIF compared to before RIF.

### 2.8. Sleep parameters

sleep duration (<7, 7–9, and > 9 hours), and sleep disturbance were the parameters assessed based on participants’ estimations before and during RF. 7–9 hours of sleep per night was considered to be optimal in duration,^[27]^ < 7 hours as short sleep duration, and > 9 h as long sleep duration, based on previously published consensus by sleep experts.^[28]^ Sleep quality was self-reported as poor, good, or very good. Participants were also asked to indicate if they experienced any of the listed sleep disturbances before and during RF: slept poorly and restlessly; hard to go to sleep; woke too early and was unable to get back to sleep; woke several times and found it difficult to get back to sleep; and no sleep disturbances. These questions were drawn from a reliable and validated instrument.^[13,24,29]^

### 2.9. Sleep quality

Three sleep quality components: sleep quality, sleep duration, and sleep disturbances were assessed based on participants’ estimations before and during RIF. Self-reported sleep quality was categorized as poor, good, or very good. Sleep duration was classified into 3 categories: <7 hours, 7–9 hours (considered optimal), and > 9 hours. Sleep disturbances included reporting items such as difficulty falling asleep, waking too early, waking multiple times, and experiencing restless sleep.

### 2.10. Sleep disturbances

The assessment of sleep disturbances in this study involved participants’ self-reported experiences before and during Ramadan fasting. Participants were asked to indicate whether they encountered specific sleep disturbances during these periods. These disturbances included sleeping poorly and restlessly, finding it hard to initiate sleep, waking too early and struggling to return to sleep, waking multiple times during the night and facing difficulty falling back asleep, and reporting no sleep disturbances. These categories were drawn from a reliable and validated instrument, providing a comprehensive understanding of the various disturbances individuals might face in their sleep patterns while fasting during Ramadan.

### 2.11. Poor sleep quality

Participants’ subjective assessment of their sleep quality played a crucial role in this study. They were asked to categorize their sleep quality as either poor, good, or very good, both before and during Ramadan fasting. This self-reported evaluation allowed for exploring how individuals perceived their sleep quality during this religious observance. Poor sleep quality, as reported by participants, served as an important parameter to consider in understanding the impact of Ramadan fasting on sleep patterns and overall well-being.

### 2.12. High/low sleep duration

The study also considered sleep duration as a vital component of participants’ sleep parameters. The researchers used established guidelines to categorize sleep duration. An optimal sleep duration of 7 to 9 hours per night was deemed ideal, aligning with expert consensus. Additionally, the study recognized sleep durations of more than 9 hours as indicative of long sleep and durations below this range as potentially insufficient. This categorization enabled the examination of how variations in sleep duration may influence the overall health and well-being of individuals observing Ramadan fasting, providing valuable insights into the relationship between sleep duration and this religious practice.

### 2.13. Statistical analysis

Descriptive statistics were used to present the demographic details of the participants, calculating frequencies and percentages to provide an overview of the sample’s demographic profile. The data’s suitability for further statistical analysis was verified through the Kolmogorov-Smirnov test, ensuring the normal distribution of data. To investigate the disruption of sleep patterns during Ramadan, we conducted a multinomial logistic regression analysis. This analysis was crucial in determining both unadjusted and adjusted odds ratios, highlighting the strength of the associations between various risk factors and changes in sleep parameters. The regression model proved invaluable in identifying the probabilities of experiencing either good or poor sleep quality throughout the fasting period. The analysis explored the relationships between several independent variables – specifically, dietary habits, physical activity (PA) levels, and sociodemographic factors – and the dependent variable of sleep parameters. The findings were reported as crude odds ratios to show direct correlations and adjusted odds ratios to consider the influence of potential confounding factors. We set a significance level at *P* < .05, with any values below this threshold considered statistically significant. All data analyses were performed using IBM SPSS Statistics for Windows, Version 23 (SPSS, Chicago).

## 3. Results

The population studied in this research included 1372 individuals. Including 509 (37.1%) men and 863 (62.9%) women. The age range of the participants was between 18-78 years with the mean and standard deviation 27.28 ± 9.80 respectively. They were based on their marital status: single (n = 931), married (n = 396), divorced (n = 38), and widowed (n = 7). They were based on their employment status: unemployed, don’t work (n = 205), student (n = 822), employed (n = 321), and retired (n-=24). They were based on their living area: city (n = 928), town (n = 308), and village (n = 136).

In Table 1, the prevalence of sleep disturbances, poor sleep quality, and unusual sleep duration is reported. The prevalence of sleep disturbances was 48% with a 95% confidence interval (CI 46–51). This prevalence for males was 39% (CI 35–44) and females 53% (CI 50–57). The prevalence of poor sleep quality was 21% with a 95% confidence interval (CI 18– 23). This prevalence for males was 15% (CI 15–21) and females 22% (CI 20–0.25). The prevalence of unusual sleep duration was 46% with a 95% confidence interval (CI 43–49). This prevalence for males was 44% (CI 40–48) and females 47% (CI 44–51). The prevalence of sleep disturbances, poor sleep quality, and unusual sleep duration based on other variables in this research is reported in detail in Table 1 based on other variables.

**Table 1:** Prevalence of sleep disturbances, Sleep quality, and sleep duration.

Sociodemographic variable	Sleep disturbances, n (%)	Poor sleep quality, n (%)	High/low sleep duration, n (%)
Total	460 (48.05)	320 (21.10)	120 (46.10)
Sex			
Male	39	15	44
Female	53	22	47
Age			
18–32	49	22	48
33–47	46	17	37
48–62	42	16	38
63≥	67	17	75
Marital status			
Single	48 % (CI 45–51)	21	47
Married	50 % (CI 45–55)	20	46
Divorced	34 % (CI 21–51)	8	21
Widowed	29 % (CI 06–70)	0	14
Body mass index (kg/m^2^)			
Underweight (*<*18.5)	59	18	60
Normal (18.50–24.9)	43% (CI 40–56)	19% (CI 17–22)	42% (CI 39–45)
Overweight (25.0–29.9)	56% (CI 49–62)	25% (CI 20–31)	47% (CI 41–54)
Obese (≥30)	62% (CI 51–71)	23 % (CI 16–33)	60% (CI 50–70)
Living area			
City	43 % (CI 40–46)	19% (CI 17–22)	42% (CI 39–45)
Town	58 % (CI 52–63)	28 % (CI 23–34)	55% (CI 49–60)
Village	64 % (CI 56–72)	14% (CI 9–21)	54% (CI 46–63)
Household income			
Lower, marginal middle (lower 20%)	62% (CI 55–68)	29% (CI 23–36)	59% (CI 52–66)
Basic middle (middle 20%)	42% (CI 38–45)	16% (CI 14–19)	40% (CI 36–43)
Upper middle (upper 20%)	51% (CI 43–58)	26% (CI 20–33)	57% (CI 49–64)
Upper (top 20%)	65% (CI 56–72)	27% (CI 20–35)	51% (CI 43–59)
Living with others			
Alone	40% (CI 30–50)	11% (CI 6–19)	31% (CI 22–41)
With friends	54% (CI 38–69)	38% (CI 24–54)	76% (CI 59–87)
With family	49% (CI 46–51)	21% (CI 19–23)	46% (CI 43–49)
Family history obesity			
Yes	59% (CI 55–62)	27% (CI 24–31)	55% (CI 51–59)
No	40% (CI 37–43)	15% (CI 13–18)	39% (CI 36–43)
Family history HTN			
Yes	56% (CI 52–59)	27% (CI 24–30)	51% (CI 47–55)
No	41% (CI 37–45)	14% (CI 12–17)	41% (CI 37–45)
Family history DM			
Yes	52% (CI 48–56)	22% (CI 19–26)	47% (CI 43–51)
No	46% (CI 42–49)	19% (CI 17–22)	45% (CI 42–49)
Family history HD			
Yes	54% (CI 51–60)	24% (CI 21–28)	49% (CI 44–53)
No	44% (CI 40–47)	18% (CI 16–21)	44% (CI 41–48)
Personal history obesity			
Yes	56% (CI 51–60)	24% (CI 20–29)	52% (CI 47–56)
No	45% (CI 42–48)	19% (CI 16–21)	43% (CI 40–47)
Personal history DM			
Yes	52% (CI 41–62)	15% (CI 9–24)	37% (CI 27–47)
No	48% (CI 45–51)	21% (CI 19–23)	47% (CI 44–49)
Personal history HTN			
Yes	62% (CI 52–71)	21% (CI 14–31)	51% (CI 41–61)
No	47% (CI 44–50)	20% (CI 18–23)	46% (CI 43–48)
Personal history HD			
Yes	64% (CI 54–72)	21% (CI 14–30)	45% (CI 35–54)
No	47% (CI 44–50)	21% (CI 18–23)	46% (CI 43–49)
Weight change			
Stable	45% (CI 41–49)	17% (CI 14–19)	42% (CI 38–45)
Loss	51% (CI 46–56)	25% (CI 21–29)	51% (CI 46–56)
Gain	54% (CI 47–62)	27% (CI 21–34)	52% (CI 45–60)
Health status			
Excellent	53% (CI 49–58)	17% (CI 14–21)	53% (CI 48–58)
Very good	33% (CI 29–37)	15% (CI 12–18)	34% (CI 31–39)
Good	59% (CI 53–65)	25% (CI 21–31)	51% (CI 45–57)
Fair	66% (CI 57–74)	37% (CI 28–46)	61% (CI 52–69)
Poor	78% (CI 58–90)	52% (CI 33–70)	52% (CI 33–70)
Diet quality			
Excellent	58% (CI 51–64)	13% (CI 9–18)	51% (CI 44–58)
Very good	30% (CI 26–35)	10% (CI 7–13)	32% (CI 28–36)
Good	55% (CI 50–59)	29% (CI 25–34)	51% (CI 46–56)
Fair	61% (CI 54–67)	30% (CI 24–37)	62% (CI 56–69)
Poor	70% (CI 59–80)	42% (CI 31–53)	51% (CI 40–63)
Physical activity			
None	56% (CI 52–59)	25% (CI 22–28)	54% (CI 51–57)
1–3 times per week	33% (CI 29–38)	14% (CI 11–18)	32% (CI 28–37)
More than 3 times per week	54% (CI 44–64)	15% (CI 9–24)	44% (CI 34–54)
Computer, works, study			
None	71% (CI 65–77)	17% (CI 13–23)	63% (CI 56–69)
Less than 30 min	57% (CI 49–65)	17% (CI 12–24)	54% (CI 46–62)
1–2 h	48% (CI 43–53)	20% (CI 16–25)	47% (CI 42–52)
3–5 h	36% (CI 32–40)	18% (CI 15–22)	34% (CI 30–38)
6+ h	51% (CI 43–58)	35% (CI 28–43)	55% (CI 47–63)
TV, computer, entertainment			
None	78% (CI 66–86)	11% (CI 5–22)	57% (CI 45–69)
Less than 30 min	58% (CI 49–67)	12% (CI 7–20)	61% (CI 52–70)
1–2 h	47% (CI 42–52)	19% (CI 16–24)	44% (CI 39–49)
3–5 h	42% (CI 39–46)	20% (CI 17–24)	39% (CI 35–43)
6+ h	54% (CI 47–62)	33% (CI 26–40)	63% (CI 55–70)
Cigarette smoking			
Nonsmoker	48% (CI 45–50)	20% (CI 18–23)	46% (CI 43–49)
Smoker	61% (CI 44–76)	28% (CI 016.–45)	50% (CI 34–66)
Shisha smoking			
Nonsmoker	48% (CI 45–50)	20% (CI 18–23)	46% (CI 43–48)
Smoker	70% (CI 51–84)	30% (CI 16–49)	63% (CI 45–79)
Education			
Primary	75% (CI 51–89)	20% (CI 8–44)	65% (CI 42–83)
High school/secondary	55% (CI 49–60)	22% (CI 17–27)	52% (CI 46–58)
Bachelor’s/under graduate	49% (CI 46–53)	22% (CI 19–25)	46% (CI 43–50)
Master’s	35% (CI 29–41)	16% (CI 12–21)	36% (CI 30–42)
PhD	50% (CI 39–61)	20% (CI 12–30)	50% (CI 39–61)
Occupation			
Unemployed	58% (CI 51–64)	13% (CI 9–19)	47% (CI 41–54)
Student	47% (CI 44–51)	23% (CI 20–26)	48% (CI 45–52)
Employed	46% (CI 41–52)	20% (CI 16–25)	40% (CI 35–46)
Retired	29% (CI 14–50)	13% (CI 4–33)	33% (CI 17–54)

There were no observations.

DM = diabetes mellitus, HD = heart disease, HTN = hypertension.

To investigate the relationship between each of the variables and sleep disturbances, poor sleep quality, and unusual sleep duration, univariate logistic regression analysis is reported in Table 2. The obtained results are shown in detail in Table 2; the most important of these findings is that women, body mass index (ref normal), place of residence town, and village (compared to the city), low and high-income levels (compared to the average), Living with friends, family history obesity, HBP, diabetic, HD, personal history obesity, weight change, Shisha smoking, and unemployment, were associated with risk of one of sleep disturbances, poor sleep quality, and unusual sleep duration, at least. On the other hand, the protective factors were: high self-reported health status, physical activity, entertainment, and self-rating diet quality.

**Table 2:** Univariate analysis of associated factors for sleep disturbances, Sleep quality, and sleep duration.

Sociodemographic variable	Sleep disturbances, n (%)	Poor sleep quality, n (%)	High/low sleep duration, n (%)
Sex			
Male	ref	ref	ref
Female	**1.74 (CI 1.40–2.18**)***	**1.33 (CI 1.008–1.76**)*	1.13 (CI 0.91–1.41)
Age			
18–32	ref	ref	ref
33–47	0.88 (CI 0.67–1.16)	0.73 (CI 0.51–1.04)	**0.62 (CI 0.47–0.82**)***
48–62	0.75 (CI 0.43–1.30)	0.70 (CI 0.33–1.45)	0.65 (CI 0.37–1.14)
63≥	2.09 (CI 0.62–6.98)	0.71 (CI 0.15–3.30)	3.19 (CI 0.86–11.87)
Marital status			
Single	ref	ref	ref
Married	1.06 (CI 0.83–1.34)	0.93 (CI 0.69–1.24)	0.97 (CI 0.77–1.23)
Divorced	0.55 (CI 0.28–1.10)	0.31 (CI 0.09–1.03)	**0.30 (CI 0.13–0.66**)**
Widowed	0.42 (CI 0.08–2.22)	–	0.18 (CI 0.02–1.56)
Body mass index (kg/m^2^)			
Underweight (*<*18.5)	**1.94 (CI 1.37–2.76**)***	0.92 (CI 0.59–1.44)	**2.10 (CI 1.48–2.98**)***
Normal (18.50–24.9)	ref	ref	ref
Overweight (25.0–29.9)	**1.66 (CI 1.24–2.21**)***	1.39 (CI 0.99–1.95)	1.26 (CI 0.94–1.68)
Obese (≥30.0)	**2.12 (CI 1.36–3.30**)***	1.24 (CI 0.74–2.07)	**2.13 (CI 1.37–3.31**)***
Living area			
City	ref	ref	ref
Town	**1.83 (CI 1.41–2.38**)***	**1.68 (CI 1.24–2.26**)***	**1.69 (CI 1.30–2.19**)***
Village	**2.38 (CI 1.64–3.46**)***	0.69 (CI 0.41–1.15)	**1.66 (CI 1.15–2.38**)**
Household income			
Lower, marginal middle (lower 20%)	**2.24 (CI 1.64–3.06**)***	**2.10 (CI 1.48–2.99**)***	**2.20 (CI 1.61–3.00**)***
Basic middle (middle 20%)	ref	ref	ref
Upper middle (upper 20%)	**1.42 (CI 1.03–1.97**)*	**1.81 (CI 1.23–2.65**)**	**1.99 (CI 1.43–2.77**)***
Upper (top 20%)	**2.56 (CI 1.76–3.72**)***	**1.85 (CI 1.22–2.82**)**	**1.58 (CI 1.10–2.27**)*
Living with others			
Alone	0.69 (CI 0.44–1.06)	**0.47 (CI 0.24–0.92**)**	**0.51 (CI 0.32–0.81**)**
With friends	1.24 (CI 0.64–2.39)	**2.32 (CI 1.18–4.58**)**	**3.61 (CI 1.69–7.73**)***
With family	ref	ref	ref
Family history obesity			
Yes	**2.12 (CI 1.71–2.64**)***	**2.03 (CI 1.56–2.65**)***	**1.86 (CI 1.15–2.32**)***
No	ref	ref	ref
Family history HTN			
Yes	**1.82 (CI 1.46–2.25**)***	**2.24 (CI 1.70–2.94**)***	**1.48 (CI 1.20–1.84**)***
No	ref	ref	ref
Family history DM			
Yes	**1.29 (CI 1.04–1.61**)*	1.18 (CI 0.90–1.54)	1.07 (CI 0.53–1.33)
No	ref	ref	ref
Family history HD			
Yes	**1.60 (CI 1.28–1.99**)**	**1.46 (CI 1.12–1.90**)**	1.19 (CI 0.96–1.48)
No	ref	ref	ref
Personal history obesity			
Yes	**1.55 (CI 1.23–1.95**)***	**1.37 (CI 1.04–1.81**)*	**1.38 (CI 1.09–1.73**)**
No	ref	ref	ref
Personal history DM			
Yes	1.16 (CI 0.75–1.79)	0.66 (CI 0.36–1.21)	0.66 (CI 0.42–1.04)
No	ref	ref	ref
Personal history HTN			
Yes	**1.85 (CI 1.21–2.82**)**	1.05 (CI 0.64–1.74)	1.24 (CI 0.82–1.87)
No	ref	ref	ref
Personal history HD			
Yes	**1.99 (CI 1.32–3.01**)***	1.02 (CI 0.62–1.67)	0.94 (CI 0.63–1.41)
No	ref	ref	ref
Weight change			
Stable	ref	ref	ref
Loss	**1.27 (CI 1.01–1.69**)*	**1.66 (CI 1.24–2.22**)***	**1.47 (CI 1.16–1.86**)***
Gain	**1.46 (CI 1.05–2,03**)*	**1.85 (CI 1.26–2.72**)***	**1.53 (CI 1.10–2.13**)*
Health status			
Excellent	**0.32 (CI 0.12–0.83**)*	**0.19 (CI 0.08–0.43**)***	1.04 (CI 0.47–2.28)
Very good	**0.13 (CI 0.05–0.35**)***	**0.16 (CI 0.07–0.36**)***	0.48 (CI 0.22–1.06)
Good	0.41 (CI 0.16–1.05)	**0.31 (CI 0.14–0.70**)**	0.96 (CI 0.43–2.12)
Fair	0.55 (CI 0.20–1.47)	0.53 (CI 0.23–1.25)	1.43 (CI 0.61–3.32)
Poor	ref	ref	ref
Self-rating diet quality			
Excellent	0.57 (CI 0.32–1.01)	**0.20 (CI 0.10–0.37**)***	0.98 (CI 0.57–1.68)
Very good	**0.18 (CI 0.10–0.31**)***	**0.14 (CI 0.8–0.25**)***	**0.44 (CI 0.27–0.72**)***
Good	**0.50 (CI 0.29–0.86**)*	**0.56 (CI 0.33–0.93**)*	0.99 (CI 0.60–1.62)
Fair	0.65 (CI 0.37–1.16)	0.59 (CI 0.34–1.02)	1.56 (CI 0.91–2.67)
Poor	ref	ref	ref
Physical activity			
None	ref	ref	ref
1–3 times per week	**0.39 (CI 0.31–0.50**)***	**0.49 (CI 0.36–0.67**)***	**0.40 (CI 0.31–0.51**)***
More than 3 times per week	0.92 (CI 0.59–1.41)	**0.53 (CI 0.29–0.97**)*	0.67 (CI 0.43–1.03)
Computer, works, study			
None	ref	ref	ref
Less than 30 min	**0.53 (CI 0.34–0.83**)**	0.98 (CI 0.56–1.72)	0.68 (CI 0.44–1.06)
1–2 h	**0.36 (CI 0.25–0.53**)***	1.17 (CI 0.74–1.85)	**0.51 (CI 0.36–0.74**)***
3–5 h	**0.22 (CI 0.15–0.31**)***	1.06 (CI 0.69–1.62)	**0.29 (CI 0.21–0.41**)***
6 + h	**0.41 (CI 0.26–0.63**)***	**2.55 (CI 1.57–4.15**)***	0.72 (CI 0.47–1.09)
TV, computer, entertainment			
None	ref	ref	ref
Less than 30 min	**0.40 (CI 0.19–0.81**)*	1.09 (CI 0.41–2.90)	1.17 (CI 0.62–2.21)
1–2 h	0.25 (CI 0.13–0.47)***	1.93 (CI 0.84–4.40)	0.58 (CI 0.34–100)
3–5 h	**0.21 (CI 0.11–0.38**)***	2.05 (CI 0.91–4.61)	**0.48 (CI 0.28–0.81**)**
6 + h	**0.34 (CI 0.17–0.66**)**	**3.85 (CI 1.65–9.02**)**	1.26 (CI 0.70–2.27)
Cigarette smoking			
Smoker	1.71 (CI 0.87–3.38)	1.50 (CI 0.71–3.15)	1.18 (CI 0.60–2.29)
Nonsmoker	ref	ref	ref
Shisha smoking			
Smoker	**2.55 (CI 1.16–5.62**)*	1.67 (CI 0.76–3.70)	2.06 (CI 0.97–4.36)
Nonsmoker	ref	ref	ref
Education			
Primary	ref	ref	ref
High school/secondary	0.40 (CI 0.14–1.13)	1.10 (CI 0.35–3.41)	0.58 (CI 0.22–1.49)
Bachelor’s/undergraduate	**0.32 (CI 0.11–0.89**)**	1.11 (CI 0.36–3.37)	0.46 (CI 0.18–1.16)
Master’s	**0.17 (CI 0.06–0.50**)***	0.76 (CI 0.24–2.39)	**0.29 (CI 0.11–0.77**)*
PhD	0.33 (CI 0.11–1.00)	0.96 (CI 0.28–3.29)	0.53 (CI 0.19–1.48)
Occupation			
Unemployed	**1.58 (CI 1.11–2.25**)*	0.59 (CI 0.36–0.97)	1.33 (CI 0.93–1.90)
Student	1.04 (CI 0.80–1.35)	1.15 (CI 0.84–1.59)	**1.39 (CI 1.07–1.80**)*
Employed	ref	ref	ref
Retired	0.48 (CI 0.19–1.19)	0.56 (CI 0.16–1.94)	0.74 (CI 0.30–1.78)

DM = diabetes mellitus, HD = heart disease, HTN = hypertension.

*< .05;

**< .01;

*** <.001.

### 3.1. Multivariate analysis

To investigate the relationship between each of the variables and sleep disturbances, Multivariate logistic regression analysis is reported in Table 3. The obtained results are shown in detail in Table 3; the most important of these findings is that women, overweight (ref normal), place of residence town (compared to the city), family history obesity, and HBP were associated with risk of sleep disturbances. On the other hand, the protective factors were: high self-reported health status, physical activity, Entertainment, retirement, and self-rating diet quality.

**Table 3:** Multivariate analysis of associated factors for sleep disturbances.

Sleep disturbances	Odds ratio	Std. Err.	z	P > z	95% Conf.	Interval
Sex						
Male	Ref					
Female	1.550231	0.2181415	3.12	**0.002**	1.176574	2.042554
Age						
18–32 years	Ref					
33–47 years	1.202471	0.2705662	0.82	0.413	0.7736543	1.868969
48–62 years	1.220171	0.4984209	0.49	0.626	0.5479214	2.717211
63 and older	3.085098	2.655358	1.31	0.191	0.5709998	16.66871
Marital status						
Single	Ref					
Married	0.8934464	0.1630097	−0.62	0.537	0.6248365	1.277529
Divorced	0.6181599	0.2816133	−1.06	0.291	0.253117	1.509664
Widowed	0.1505638	0.2065342	−1.38	0.168	0.0102352	2.214861
BMI						
Underweight	1.346296	0.2764266	1.45	0.148	0.900261	2.013318
Normal weight	Ref					
Overweight	1.479205	0.2838247	2.04	**0.041**	1.015554	2.154537
Obese	1.473563	0.3970873	1.44	0.150	0.8689439	2.498881
Living area						
City	Ref					
Town	1.37045	0.2074034	2.08	**0.037**	1.018692	1.843673
Village	1.413924	0.3198774	1.53	0.126	0.9075185	2.202909
House hold income						
Lower, marginal middle 20%	1.336118	0.2406929	1.61	0.108	0.9386564	1.901881
Basic middle 20%	Ref					
Upper middle 20%	1.109263	0.2057997	0.56	0.576	0.7711033	1.59572
Upper class 20%	1.523588	0.3353377	1.91	0.056	0.9897381	2.345388
With whom living						
Alone	1.082746	0.3368059	0.26	0.798	0.5884996	1.99208
With friends	1.047064	0.3980903	0.12	0.904	0.4969915	2.205958
With family	Ref					
Family history obesity						
Yes	1.397406	0.2177319	2.15	**0.032**	1.029664	1.896485
No	Ref					
Family history HTN						
Yes	1.413982	0.2094061	2.34	**0.019**	1.057752	1.890184
No	Ref					
Family history DM						
Yes	1.085344	0.1562801	0.57	0.570	0.8184679	1.439239
No	Ref					
Family history HD						
Yes	1.262671	0.1917331	1.54	0.125	0.9376436	1.700368
No	Ref					
Personal history obesity						
Yes	0.7657401	0.1365203	−1.50	0.134	0.539914	1.086021
No	Ref					
Personal history DM						
Yes	0.9373383	0.2805253	−0.22	0.829	0.5213747	1.685166
No	Ref					
Personal history HTN						
Yes	1.181287	0.3523919	0.56	0.577	0.658312	2.119722
No	Ref					
Personal history HD						
Yes	1.450466	0.390214	1.38	0.167	0.8560748	2.457556
No	Ref					
Weight gain						
Stable	Ref					
Loss	1.087914	0.1518173	0.60	0.546	0.8275806	1.430141
Gain	1.171696	0.2272735	0.82	0.414	0.8011383	1.71365
Health status						
Excellent	0.4538176	0.2384952	−1.50	0.133	0.1620125	1.2712
Very good	0.292309	0.153129	−2.35	**0.019**	0.1046964	0.8161173
Good	0.5481573	0.285932	−1.15	0.249	0.197196	1.523745
Fair	0.6154519	0.3355593	−0.89	0.373	0.2113968	1.791801
Poor	Ref					
Self-rating diet quality						
Excellent	0.9236761	0.3130011	−0.23	0.815	0.4754202	1.794575
Very good	0.5128219	0.1644625	−2.08	**0.037**	0.2735176	0.9614966
Good	0.828725	0.2551307	−0.61	0.542	0.4532733	1.515168
Fair	0.8037116	0.2595102	−0.68	0.499	0.4268311	1.513368
Poor	Ref					
Physical activity						
None	Ref					
1–3 times per week	0.6315105	0.089856	−3.23	**0.001**	0.4778211	0.8346335
More than 3 times per week	1.029902	0.2486712	0.12	0.903	0.6416114	1.653178
Computer work-study						
None	Ref					
Less than 30 min	0.7305033	0.1866508	−1.23	**0.219**	0.4427227	1.205348
1–2 h	0.5898626	0.1311885	−2.37	**0.018**	0.3814515	0.912142
3–5 h	0.4908334	0.107861	−3.24	**0.001**	0.3190671	0.7550683
6 + h	0.5219176	0.1327326	−2.56	**0.011**	0.3170493	0.8591659
TV computer entertainment during						
None	Ref					
Less than 30 min	0.6382319	0.256577	−1.12	0.264	0.2902587	1.403369
1–2 hours	0.4128873	0.1491825	−2.45	**0.014**	0.2033665	0.8382694
3–5 hours	0.4909606	0.1761661	−1.98	**0.047**	0.2430072	0.9919141
6 + hours	0.4189902	0.1612619	−2.26	**0.024**	0.1970569	0.8908739
Smoking						
Nonsmoker	Ref					
Smoker	1.182121	0.479736	0.41	0.680	0.5336072	2.618798
Shisha smoking						
None smoker	Ref					
Smoker	2.076145	0.93206	1.63	0.104	0.8612341	5.004886
Education						
Primary	Ref					
High school or secondary	0.7151577	0.43685	−0.55	0.583	0.2159982	2.367846
BSc undergraduate	0.6497355	0.392791	−0.71	0.476	0.1986787	2.124819
MSc	0.4392074	0.2738341	−1.32	0.187	0.1294101	1.490634
PhD	0.5965552	0.3826389	−0.81	0.421	0.1696985	2.09712
Occupation						
Unemployed, don’t work	0.7846456	0.1877592	−1.01	0.311	0.4908943	1.254178
Student	0.6475113	0.1341191	−2.10	**0.036**	0.4314573	0.9717553
Employed	1	(base)				
Retired	0.2199011	0.1474011	−2.26	**0.024**	0.0591101	0.818075

DM = diabetes mellitus, HD = heart disease, HTN = hypertension.

To investigate the relationship between each of the variables and poor sleep quality, Multivariate logistic regression analysis is reported in Table 4. The obtained results are shown in detail in Table 4; the most important of these findings is that living with friends, family history HBP, and lower and upper Household Income (ref basic) were associated with the risk of sleep quality. On the other hand, the protective factors were: high self-reported health status and self-rating diet quality.

**Table 4:** Multivariate analysis of associated factors for poor sleep quality.

Sleep quality	Odds ratio	Std. Err.	z	P > z	95% Conf.	Interval
Sex						
Male	Ref					
Female	1.309705	0.2258121	1.56	0.118	0.9341444	1.836255
Age						
18–32 yr	Ref					
33–47 yr	0.9305201	0.2546007	−0.26	0.792	0.5442864	1.590831
48–62 yr	0.7451148	0.3873699	−0.57	0.571	0.2689682	2.06417
63 and older	0.6913031	0.7214142	−0.35	0.724	0.0894111	5.344977
Marital status						
Single	Ref					
Married	1.303956	0.2850346	1.21	0.225	0.8495653	2.001377
Divorced	0.4513315	0.3211639	−1.12	0.264	0.111889	1.820556
Widowed	1	(empty)				
BMI						
Underweight	0.7344965	0.19289	−1.17	0.240	0.4389866	1.228933
Normal weight	Ref					
Overweight	1.168172	0.2583656	0.70	0.482	0.7572615	1.802054
Obese	0.8467401	0.2712678	−0.52	0.604	0.4519111	1.586526
Living area						
City	Ref					
Town	1.261276	0.2191787	1.34	0.182	0.8972064	1.773079
Village	0.6202828	0.1872797	−1.58	0.114	0.3432334	1.12096
House hold income						
Lower, marginal middle 20%	1.674512	0.3358381	2.57	**0.010**	1.130244	2.480871
Basic middle 20%	Ref					
Upper middle 20%	1.582842	0.3441837	2.11	**0.035**	1.033586	2.423977
Upper class 20%	2.165658	0.5559497	3.01	0.003	1.309412	3.581816
With whom living						
Alone	0.8108961	0.3244471	−0.52	0.600	0.3701617	1.776392
With friends	2.405672	0.9421171	2.24	**0.025**	1.11657	5.183066
With family	Ref					
Family history obesity						
Yes	1.133791	0.2097069	0.68	0.497	0.7890307	1.629191
No	Ref					
Family history HTN						
Yes	1.844984	0.3323098	3.40	**0.001**	1.296218	2.626074
No	Ref					
Family history DM						
Yes	0.8630703	0.1460851	−0.87	0.384	0.6193983	1.202603
No	Ref					
Family history HD						
Yes	1.043509	0.184097	0.24	0.809	0.738459	1.474573
No	Ref					
Personal history obesity						
Yes	0.8407739	0.1788995	−0.82	0.415	0.5540653	1.275844
No	Ref					
Personal history DM						
Yes	0.8778671	0.3281013	−0.35	0.727	0.4219817	1.826265
No	Ref					
Personal history HTN						
Yes	1.417147	0.4717121	1.05	0.295	0.7380465	2.721111
No	Ref					
Personal history HD						
Yes	0.7602791	0.2350184	−0.89	0.375	0.4148094	1.39347
No	Ref					
Weight gain						
Stable	Ref					
Loss	1.282415	0.2141073	1.49	0.136	0.9245174	1.778861
Gain	1.348371	0.2996502	1.34	0.179	0.8722594	2.084361
Health status						
Excellent	0.2835319	0.1368436	−2.61	**0.009**	0.1100978	0.730172
Very good	0.2720758	0.1304254	−2.72	**0.007**	0.1063281	0.6961963
Good	0.2873554	0.1362366	−2.63	**0.009**	0.1134646	0.7277434
Fair	0.3958519	0.196232	−1.87	0.062	0.1498216	1.045902
Poor	Ref					
Self-rating diet quality						
Excellent	0.3859586	0.1430818	−20.57	**0.010**	0.1866316	0.7981719
Very good	0.3242678	0.1100162	−30.32	**0.001**	0.1667677	0.6305156
Good	0.9840167	0.2986623	−0.05	0.958	0.5428144	1.78383
Fair	0.8206604	0.2590363	−0.63	0.531	0.4420659	1.523491
Poor	Ref					
Physical activity						
None	Ref					
1–3 times per week	0.7215713	0.1291516	−1.82	0.068	0.5080724	1.024785
More than 3 times per week	0.5921484	0.1943928	−1.60	0.110	0.3111678	1.126851
Computer work-study						
None	Ref					
Less than 30 min	1.117779	0.3596229	0.35	0.729	0.5949758	2.099969
1–2 h	1.222316	0.3351634	0.73	0.464	0.7141362	2.092117
3–5 h	1.524327	0.4064785	1.58	0.114	0.9038488	2.570753
6 + h	2.236331	0.6552516	2.75	**0.006**	1.259306	3.971372
TV computer entertainment during						
None	Ref					
Less than 30 min	0.7549332	0.4188355	−0.51	0.612	0.2544858	2.239513
1–2 h	1.193114	0.5601539	0.38	0.707	0.4753923	2.994413
3–5 h	1.527623	0.7073288	0.92	0.360	0.6164361	3.785685
6 + h	1.793776	0.868319	1.21	0.227	0.6945832	4.632464
Smoking						
Nonsmoker	Ref					
Smoker	1.417632	0.6604597	0.75	0.454	0.5688497	3.532883
Shisha smoking						
None smoker	Ref					
Smoker	1.713673	0.7663514	1.20	0.228	0.7132996	4.117027
Education						
Primary	Ref					
High school or secondary	0.4341773	0.3114859	−1.16	0.245	0.1064146	1.771467
BSc undergraduate	0.4011026	0.2848023	−1.29	0.198	0.0997381	1.613057
MSc	0.3556778	0.2601426	−1.41	0.158	0.0848181	1.491506
PhD	0.3095281	0.2364026	−1.54	0.125	0.0692777	1.382951
Occupation						
Unemployed, don’t work	0.5184865	0.159355	−2.14	0.033	0.2838727	0.9470027
Student	1.12487	0.2698716	0.49	0.624	0.7028903	1.800185
Employed	Ref					
Retired	0.7599385	0.6371537	−0.33	0.743	0.1469289	3.930517

DM = diabetes mellitus, HD = heart disease, HTN = hypertension.

**Figure 1.**
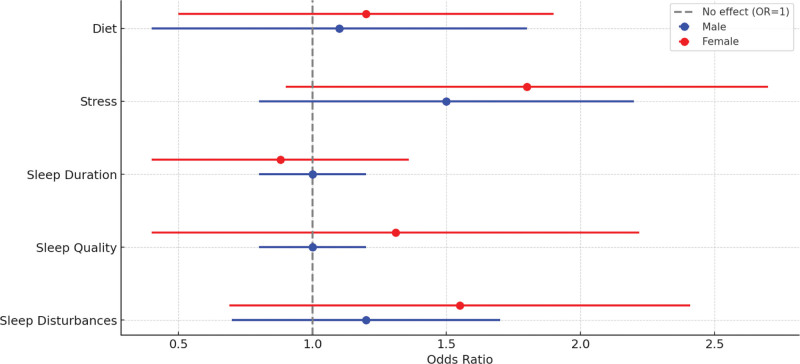
Odds ratios for sleep issues and associated factors by sex.

The forest plot comprehensively presents the odds ratios for various sleep issues and associated factors across genders. Females were found to have statistically significantly higher odds of experiencing sleep disturbances (OR = 1.55), poor sleep quality (OR = 1.31), and stress (OR = 1.8) compared to males (Fig. 1).

In contrast, females had slightly lower odds of abnormal sleep duration (OR = 0.88). Males showed no significant risk for poor sleep quality and abnormal sleep duration but had higher odds for stress (OR = 1.5) and diet-related issues (OR = 1.1). All findings were statistically significant as indicated by confidence intervals not overlapping with 1.

Multivariate logistic regression analysis is reported in Table 5. The obtained results are shown in detail in Table 5. Age 63 and older, underweight (ref normal weight), lower and upper Household Income (ref basic), living with friends, and family history HBP were associated with a risk of unusual sleep duration. On the other hand, the protective factor was: physical activity.

**Table 5:** Multivariate analysis of associated factors for sleep duration.

Sleep duration	Odds ratio	Std. Err.	z	P > z	95% Conf.	Interval
Sex						
Male	Ref					
Female	0.8766514	0.1207164	−0.96	0.339	0.669291	1.148256
Age						
18–32 yr	Ref					
33–47 yr	0.8125671	0.1759466	−0.96	0.338	0.5315536	1.242143
48–62 yr	1.222617	0.4895083	0.50	0.616	0.5578133	2.679738
63 and older	7.186713	6.290947	2.25	**0.024**	1.292481	39.96101
Marital status						
Single	Ref					
Married	1.211821	0.2145594	1.09	0.278	0.8565026	1.714543
Divorced	0.603961	0.2966396	−1.03	0.305	0.2306404	1.581548
Widowed	0.1707033	0.2459002	−1.23	0.220	0.0101409	2.87348
BMI						
Underweight	1.731296	0.3512135	2.71	**0.007**	1.163309	2.576602
Normal weight	1	(base)				
Overweight	0.9509237	0.1765717	−0.27	0.786	0.6608316	1.36836
Obese	1.433159	0.3765325	1.37	0.171	0.8563655	2.398445
Living area						
City	Ref					
Town	1.194187	0.1765105	1.20	0.230	0.8938365	1.595464
Village	1.037369	0.227893	0.17	0.867	0.6744324	1.595616
House hold income						
Lower, marginal middle 20%	1.427609	0.2477364	2.05	**0.040**	1.016011	2.005951
Basic middle 20%	Ref					
Upper middle 20%	1.532729	0.2818776	2.32	**0.020**	1.068869	2.197891
Upper class 20%	1.002401	0.2140329	0.01	0.991	0.6596184	1.523317
With whom living						
Alone	.9131955	0.2735726	−0.30	0.762	0.5076485	1.642723
With friends	3.597994	1.49238	3.09	**0.002**	1.595872	8.111903
With family	Ref					
Family history obesity						
Yes	1.109349	0.1707202	0.67	0.500	0.8204928	1.499897
No	Ref					
Family history HTN						
Yes	1.423349	0.210257	2.39	**0.017**	1.065545	1.9013
No	Ref					
Family history DM						
Yes	.9440776	0.1342434	−0.40	0.686	0.7144483	1.247512
No	Ref					
Family history HD						
Yes	1.116075	0.1680335	0.73	0.466	0.8308803	1.499161
No	Ref					
Personal history obesity						
Yes	1.057497	0.1849336	0.32	0.749	0.7506233	1.489828
No	Ref					
Personal history DM						
Yes	.7094794	0.2103481	−1.16	0.247	0.3968017	1.268545
No	Ref					
Personal history HTN						
Yes	1.126887	0.3259347	0.41	0.680	0.6392683	1.986448
No	Ref					
Personal history HD						
Yes	.6882591	0.1792882	−1.43	0.152	0.4130647	1.146795
No	Ref					
Weight gain						
Stable	Ref					
Loss	1.207949	0.1647802	1.38	0.166	0.9245569	1.578206
Gain	1.246322	0.2357517	1.16	0.244	0.860238	1.805684
Health status						
Excellent	1.377321	0.624663	0.71	0.480	0.5662206	3.350308
Very good	.9620176	0.4342909	−0.09	0.932	0.3971172	2.330491
Good	1.144104	0.5131752	0.30	0.764	0.4749724	2.755894
Fair	1.624239	0.7695564	1.02	0.306	0.641734	4.110977
Poor	Ref					
Self–rating diet quality						
Excellent	1.456028	0.4657732	1.17	0.240	0.7778154	2.725607
Very good	1.082409	0.3283356	0.26	0.794	0.5972959	1.961521
Good	1.529453	0.4401921	1.48	0.140	0.8700654	2.688566
Fair	1.941954	0.5878447	2.19	**0.028**	1.072935	3.51483
Poor	Ref					
Physical activity						
None	Ref					
1–3 times per week	0.5433036	0.0764847	−4.33	**0.000**	0.4122996	0.7159327
More than 3 times per week	0.6433283	0.1545173	−1.84	0.066	0.4017792	1.030096
Computer work-study						
None	Ref					
Less than 30 min	0.6744759	0.1665007	−1.60	0.111	0.4157568	1.094192
1–2 h	0.6482165	0.1387232	−2.03	**0.043**	0.4261442	0.9860153
3–5 h	0.4575454	0.0968999	−3.69	**0.000**	0.30211	0.6929524
6 + h	0.6916097	0.1711082	−1.49	0.136	0.425862	1.12319
TV computer entertainment						
None	Ref					
Less than 30 min	1.644569	0.6023007	1.36	0.174	0.8022532	3.371265
1–2 h	0.8500424	0.2720672	−0.51	0.612	0.4539441	1.591765
3–5 h	0.9173238	0.2907975	−0.27	0.785	0.4928177	1.707493
6 + h	1.381181	0.4795066	0.93	0.352	0.6994198	2.727492
Smoking						
Nonsmoker	Ref					
Smoker	0.8559314	0.3380578	−0.39	0.694	0.3946837	1.856217
Shisha smoking						
None smoker	Ref					
Smoker	1.490976	0.6275654	0.95	0.343	0.6534193	3.402116
Education						
Primary	Ref					
High school or secondary	0.7976938	0.4547095	−0.40	0.692	0.2609911	2.438073
BSc undergraduate	0.6653384	0.3757586	−0.72	0.471	0.2199456	2.012657
MSc	0.7682992	0.4484859	−0.45	0.652	0.2447097	2.412179
PhD	0.7129447	0.4285771	−0.56	0.574	0.2194661	2.31603
Occupation						
Unemployed, don’t work	0.9228725	0.2129406	−0.35	0.728	0.5871354	1.450592
Student	1.109547	0.2209759	0.52	0.602	0.7509685	1.639344
Employed	Ref					
Retired	0.3546931	0.2350942	−1.56	0.118	0.0967537	1.300283

DM = diabetes mellitus, HD = heart disease, HTN = hypertension.

## 4. Discussion

In our study conducted with 1372 participants in Iran during Ramadan amid the COVID-19 pandemic, we observed significant sleep disturbances. Specifically, 48% of participants reported sleep disturbances, 21% experienced poor sleep quality, and 46% faced irregular sleep durations. The analysis identified several factors influencing sleep patterns, including body mass index, residential location, income level, cohabitation status, family and personal history of obesity, hypertension, diabetes, heart disease, weight fluctuations, Shisha smoking, and unemployment status. These determinants played a critical role in the sleep disruptions observed among the study population. These findings are consistent with a growing body of literature highlighting the widespread impact of the pandemic on sleep health. For example, a study by Jahrami et al^[30]^ across 45 countries reported a sleep disturbance prevalence of 40.49%, emphasizing the global scale of the issue. Similarly the prevalence of sleep disturbances reported by Tedjasukmana et al^[31]^, who found a rate of 78.58% in post-COVID-19 conditions highlighting the long-lasting and severe health implications of the pandemic, extending beyond the period of active infection.

Our study was conducted during Ramadan, a period known to influence sleep behaviors Akbari et al^[15]^ reported that individuals who fasted during Ramadan experienced better sleep quality, potentially explaining the lower prevalence of sleep disturbances in our study compared to the 73.5% reported by Torkian et al. Beyond these temporal factors, our study also identified a range of lifestyle and health-related variables, such as poor diet, lack of physical activity, and older age, as significant contributors to sleep issues. Notably, unlike the study by Torkian et al^[32]^, which linked low income with poor sleep, our findings indicate that both high and low-income levels can contribute to sleep problems. These findings underscore the complex, perhaps non-linear, relationships between socioeconomic status and sleep quality. Further research is needed to understand these dynamics better, especially how they interact with other variables like stress and access to healthcare.

Moreover, our findings corroborate and extend existing literature concerning lifestyle and health-related variables affecting sleep. For example, we found that factors like self-rating poor diet quality, lack of physical activity, and old age contribute to sleep disturbances. Interestingly, despite Behbahani et al reporting a positive association between diet quality and sleep, our study found lower sleep quality even among those who reported an “excellent” diet.^[33,34]^ This incongruity suggests that the relationship between self-rating diet quality and sleep may be more complex and modulated by other unaccounted-for variables.^[35]^

Our study also aligns with previous work showing the increased prevalence of sleep issues among healthcare workers^[36]^ and the role of family social support in sleep quality.^[37]^ Given the multifaceted nature of sleep disturbances, it becomes clear that a singular focus on any one factor would be inadequate for understanding, let alone addressing, sleep problems during a pandemic.

The psychological repercussions of the pandemic further complicate the issue. Our study echoes existing research.^[38,39]^ in pointing out the intertwined nature of sleep disturbances with psychological disorders. Indeed, it seems that the pandemic has both direct and indirect effects on sleep, mediated through psychological stress.^[40–42]^ This dual impact necessitates a more integrated approach to healthcare solutions.

In summary, our study adds a deeper understanding of the complexity of sleep health during the COVID-19 pandemic. Given the multifactorial nature of sleep disturbances, a comprehensive, multi-disciplinary approach is essential for both further research and healthcare interventions. One of the key strengths of this study is its relatively large sample size of 1372 participants. Moreover, the timing of the study during Ramadan allows for unique insights into the influence of religious and cultural practices on sleep quality, a topic underexplored in existing literature. Additionally, the study’s comprehensive approach to identifying a wide range of lifestyle and health-related variables affecting sleep contributes to a more holistic understanding of sleep health.

Despite its contributions, the study is not without limitations. First, the cross-sectional design precludes any causal inferences between the identified variables and sleep disturbances. Longitudinal studies are needed to establish causality. Second, the reliance on self-reported and self-rating measures introduces the potential for reporting bias. Future research could benefit from incorporating objective measures like polysomnography. Third, while the study offers insights into the Iranian population during Ramadan, the generalizability of these findings to other cultural or temporal contexts remains uncertain. Though we acknowledge the limitations of our sampling method, the comprehensive analysis and the significant sample size provide valuable insights into sleep disturbances during Ramadan amid the COVID-19 pandemic in Iran. We suggest future research to include a detailed comparison with national demographic data to further validate the representativeness and generalizability of the findings.

The findings of our study reveal a high prevalence of sleep disturbances in Iran during Ramadan amid the COVID-19 pandemic, emphasizing the complex interplay of individual characteristics and lifestyle choices in sleep patterns. These disturbances, influenced by cultural and global health challenges, necessitate tailored interventions focused on sleep-related concerns in such unique contexts. Based on the current research it is necessary to make wider efforts in improving the quality of sleep, including prevention, lifestyle modifications, and education, at the level of the general population. It is also necessary for health professionals to pay more attention to the issue of sleep disturbances, as its effects on other dimensions of life can be extensive and challenge a person’s life.

Our study highlights the importance of health education and promotion strategies that consider individual and cultural intricacies, particularly in managing stress and lifestyle adaptations during times of global health crises. Further research, especially in diverse cultural settings, is essential to develop comprehensive health promotion programs that effectively address sleep disturbances in similar situations.

Our study’s findings underscore the urgent need for longitudinal research to explore the multifactorial nature of sleep disturbances, particularly during public health crises like the COVID-19 pandemic. Future studies could benefit from incorporating objective sleep measures alongside self-reported data to provide a more comprehensive picture of sleep health. Moreover, research that examines the interaction between cultural, economic, and psychosocial factors can offer invaluable insights into sleep disturbances on both a societal and individual level.

The high prevalence of sleep disturbances found in our study suggests an immediate need for healthcare providers to consider sleep health as an integral part of public health planning. Given the complex interplay of factors influencing sleep, a multi-disciplinary approach that incorporates lifestyle modification, mental health support, and perhaps even socioeconomic interventions is warranted. Healthcare providers should also be trained to recognize the signs of sleep disturbances and should consider screening for sleep issues as part of routine medical evaluations, especially during times of widespread public health crises. These measures could facilitate early intervention, thereby mitigating the long-term impact of sleep disturbances on quality of life and overall well-being.

## 5. Conclusion

In conclusion, this study emphasizes the pervasive issue of sleep disturbances in the Iranian population during the COVID-19 pandemic, revealing significant prevalence rates of sleep disruptions, poor sleep quality, and irregular sleep duration. These findings resonate with global trends linking infectious diseases like COVID-19 to compromised sleep health. Multiple variables, ranging from lifestyle decisions and health conditions to age and prolonged computer use, contribute to these sleep issues. Importantly, the study highlights the interrelatedness of sleep disturbances with psychological stress exacerbated by the pandemic. This co-occurrence of mental illnesses and sleep issues magnifies the adverse impact of global health crises on individual well-being. Given these implications, healthcare providers should prioritize sleep health as an urgent public health issue. Extensive initiatives focusing on prevention, lifestyle modifications, and education are imperative for improving sleep quality across the population. The significant negative effects of sleep disturbances warrant immediate attention for the overall health and well-being of the community, especially during global crises such as the COVID-19 pandemic.

## Acknowledgments

We express our gratitude to the dedicated researchers Amir Human Hoveidaei, Zeinab Tavaloli Fard, and Golnaz Kheirandish Holm for their valuable assistance in data collection.

## Author contributions

**Conceptualization:** Sohrab Amiri, MoezAlIslam E. Faris, Sajjad Ahmed Khan, Moien A.B. Khan.

**Data curation:** Sohrab Amiri.

**Formal analysis:** Mohammad Pourfridoni, Reza Heidari-Soureshjani, Mitra Sotoudeh, Amna G. Albalushi, Fatima Alsaedi.

**Investigation:** Mohammad Pourfridoni, Reza Heidari-Soureshjani, Mitra Sotoudeh, Amna G. Albalushi, Fatima Alsaedi.

**Methodology:** Sohrab Amiri.

**Writing – original draft:** Sohrab Amiri, Moien A.B. Khan.

**Writing – review & editing:** MoezAlIslam E. Faris, Sajjad Ahmed Khan, Moien A.B. Khan.
